# Plant parasitic nematode effectors target host defense and nuclear functions to establish feeding cells

**DOI:** 10.3389/fpls.2013.00053

**Published:** 2013-03-13

**Authors:** Michaëel Quentin, Pierre Abad, Bruno Favery

**Affiliations:** Institut Sophia Agrobiotech, UMR INRA 1355 – Université Nice-Sophia Antipolis – CNRS 7254Sophia Antipolis, France

**Keywords:** root-knot nematodes, cyst nematodes, effectors, plant nuclei, feeding cells

## Abstract

Plant parasitic nematodes are microscopic worms, the most damaging species of which have adopted a sedentary lifestyle within their hosts. These obligate endoparasites have a biotrophic relationship with plants, in which they induce the differentiation of root cells into hypertrophied, multinucleate feeding cells (FCs). Effectors synthesized in the esophageal glands of the nematode are injected into the plant cells via the syringe-like stylet and play a key role in manipulating the host machinery. The establishment of specialized FCs requires these effectors to modulate many aspects of plant cell morphogenesis and physiology, including defense responses. This cell reprogramming requires changes to host nuclear processes. Some proteins encoded by parasitism genes target host nuclei. Several of these proteins were immunolocalized within FC nuclei or shown to interact with host nuclear proteins. Comparative genomics and functional analyses are gradually revealing the roles of nematode effectors. We describe here these effectors and their hypothesized roles in the unique feeding behavior of these pests.

## INTRODUCTION

**FIGURE 1:**
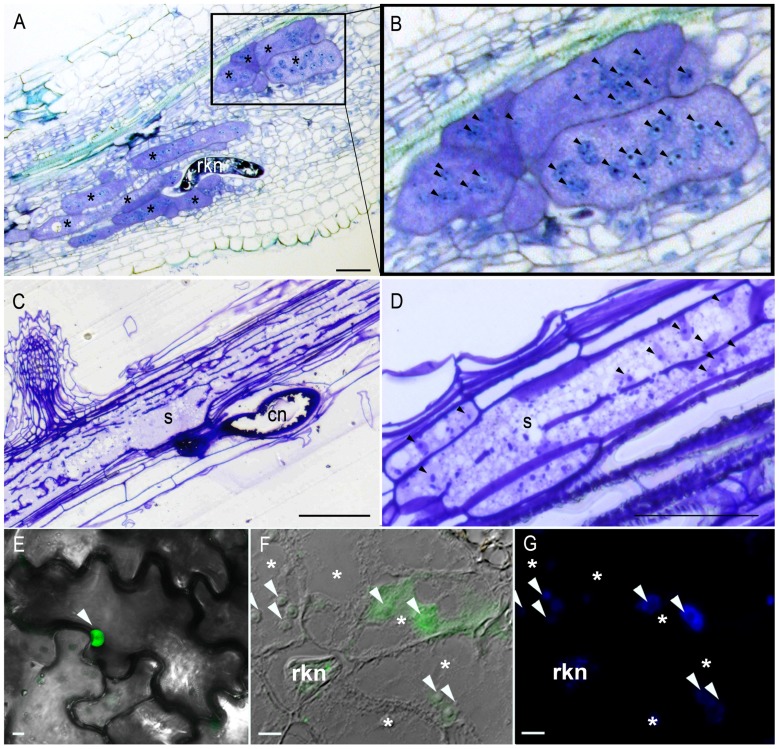
**Multinucleate and hypertrophied feeding cells induced by endoparasitic plant nematodes and nuclear localization of a RKN effector *in planta*.** **(A)** Giant cells induced by the root-knot nematode *Meloidogyne chitwoodi *in pepper. **(B)** Multiple nuclei (arrowheads) are visible in the giant cells. **(C,D)** Syncytium induced by the cyst nematode *H. schachtii *in *Arabidopsis*. Cell wall dissolution results in a multinucleate cell. Toluidine blue-stained **(A,B)** or Crystal Violet-stained **(C,D)** longitudinal sections of infected roots 7 **(A,B)** or 10 **(C,D)** days after inoculation. **(E)** RKN effector MiEFF1::GFP accumulates in the nucleus (arrowhead) of tobacco epidermal leaf cells after agroinfiltration. **(F)** Immunolocalization of the secreted MiEFF1 in tomato galls 14 days after inoculation. FITC signal is observed at the tip of the stylet of a sedentary parasitic juvenile and in the nuclei (arrowheads) of giant cells. **(G) **DAPI-staining of nuclei of the section presented in **(F)**. rkn, root-knot nematode; *, giant cells; s, syncytium; cn, cyst nematode. Bars = 50 μm **(A–D)** or 10 μm **(E–G)**.

Plant parasitic nematodes (PPNs) are small roundworms comprising about 4,000 species infesting roots of thousands of plant species and causing tremendous crop yield losses worldwide (Blok et al., 2008). The sedentary endoparasites, root-knot nematodes, (RKNs, *Meloidogyne* spp.) and cyst nematodes (CNs, *Globodera* spp. and *Heterodera* spp.), are among the most economically damaging PPNs. These parasites are obligate biotrophs that can feed only on the cytoplasm of living cells. Thus, both RKNs and CNs establish an intimate relationship with their host plants, inducing the redifferentiation of root cells into specialized multinucleate feeding cells (FCs). RKNs cause the formation and maintenance of five to seven giant cells, whereas CNs induce a syncytium (**Figure 1**). The first sign of giant cell induction by RKNs is the formation of one or several binucleate cells. These cells then go on to become multinucleate, through repeated nuclear divisions (karyokinesis) without cell division (Caillaud et al., 2008b). The hyperplasia and hypertrophy of the surrounding cells lead to the formation of the typical gall. In syncytia, the initial FC expands into the vascular tissue by the progressive and local dissolution of cell walls, resulting in the fusion of hundreds of neighboring root cells (Sobczak and Golinowski, 2011).

Fully differentiated FC is several hundred times the size of a normal root vascular cell. The cell walls thicken and ingrowths develop, facilitating solute exchange across the FC plasma membrane and sustaining nematode feeding until adult stages. Within the dense cytoplasm of the developing FC, subcellular organelles proliferate, their nuclei and nucleoli enlarge, and small secondary vacuoles are formed (**Figure 1**). FCs constitute the sole source of nutrients for the nematodes and are essential for their growth and reproduction. The complex changes in cellular morphology and physiology leading to FC establishment result from extensive changes to gene expression in the infected root cells. Patterns of host gene transcription have been compared by various techniques (Gheysen and Fenoll, 2002; Caillaud et al., 2008a), including, in particular, the recent genome-wide expression profiling of isolated giant cells or syncytia (Szakasits et al., 2009; Barcala et al., 2010; Damiani et al., 2012). These studies have led to the identification of many genes involved in diverse processes, such as cell cycle activation, cell wall modification, hormone and defense responses that are differentially expressed in FC formation. It remains unclear how this developmental switch allowing the nematodes to settle and resulting in changes to root cell morphology and the induction of FC occurs. However, it is now widely accepted that secreted nematode effectors play key roles in parasitism.

One of the characteristic features of PPNs is the presence of specialized esophageal gland cells allowing the production of proteins that are then secreted into the host through a hollow protrusible syringe-like stylet. The activity of these esophageal glands is developmentally regulated. Two subventral glands are particularly active during the preparasitic stages, secreting proteins involved in root invasion and larva migration, whereas a dorsal gland becomes hypertrophied and actively secretes effectors responsible for FC initiation and maintenance during the sedentary stages (Davis et al., 2008). In addition, proteins thought to be involved in parasitism are secreted into the apoplasm through the cuticle or via the chemosensory organs, the amphids. Various strategies have been used to identify nematode effectors. Proteomic approaches have been applied to purified *M. incognita* secretions (Bellafiore et al., 2008). Transcriptomic approaches, benefiting from recent advances in next-generation sequencing technologies, have made it possible to generate CN and RKN expressed sequence tags (ESTs) from various juvenile developmental stages (Roze et al., 2008; Jones et al., 2009; Jaouannet et al., 2012; Haegeman et al., 2013), infected plant tissues (Haegeman et al., 2013), or microaspiration of the cytoplasmic content of the esophageal glands (Wang et al., 2001; Gao et al., 2003; Huang et al., 2004) or isolated whole glands (Maier et al., 2013). Finally, comparative genomics approaches have facilitated a major breakthrough in effector identification. Two RKN genomes are now available, for *M. incognita* and *M. hapla* (Abad et al., 2008; Opperman et al., 2008) and increasing amounts of genomic information are being released for the soybean CN *H. glycines* and the potato CN *G. pallida*. The increasing availability of such data has led to the prediction of large effector repertoires. *In situ* hybridization studies have confirmed the specific expression of several of these putative effectors in the esophageal glands, suggesting their probable secretion into the host via the stylet and, thus, a role in infection. The identification of these effectors has made it possible to initiate functional analyses, which should make it possible to decipher the roles of these proteins in the targeting and manipulation of host functions (Haegeman et al., 2012; Hewezi and Baum, 2013). In this review, we describe nematode effectors that interact with host proteins or mimic host proteins, manipulating various aspects of plant physiology, including plant defense responses, and others that are targeted to the nucleus, where they may manipulate the nuclear machinery or bind to nucleotides (**Table 1**).

**Table 1:** Nematode effectors mentioned in this review, that target host functions to establish feeding cells.

Effector	Predicted function	Host function	Identified plant target	Reference
***Globodera* spp.**
GpRBP-1	SPRYSEC	Defense	NB-LRR-resistant protein potato GPA-2	Sacco et al. (2009)
GpCM	Chorismate mutase	Hormone and/or defense	–	Jones et al. (2003)
GrVAP1	Venom allergen protein	Defense	Papain-like cysteine protease Rcr3^pim^	Lozano-Torres et al. (2012)
GrSPRYSEC-19	SPRYSEC	Defense	NB-LRR protein tomato SW5F	Rehman et al. (2009)
GrCLE1	CLE-like peptide	Hormone	Receptors AtCLV2 and AtBAM1 and 2	Guo et al. (2011)
***Heterodera* spp.**
HgSYV46	CLE-like peptide	Hormone	–	Wang et al. (2005)
Hg30C02	Unknown	Defense	β-1,3-endoglucanase	Hamamouch et al. (2012)
HgCM	Chorismate mutase	Hormone and/or defense	–	Bekal et al. (2003)
HsCM	Chorismate mutase	Hormone and/or defense	–	Vanholme et al. (2009)
HsCBP	Cellulose-binding protein	Cell wall	Pectin methylesterase AtPME3	Hewezi et al. (2008)
HsCLE1 and 2	CLE-like peptide	Hormone	–	Wang et al. (2011)
Hs19C07	Unknown	Hormone	Plasma membrane auxin influx transporter AtLAX3	Lee et al. (2011)
Hs10A06	Unknown	Defense	Spermidine synthase AtSPDS2	Hewezi et al. (2010)
Hs4F01	Annexin-like	Defense	Oxidoreductase of the 2OG-Fe(II) oxygenase family	Patel et al. (2010)
HsUbiI	Ubiquitin extension protein	Synthesis	–	Tytgat et al. (2004)
***Meloidogyne* spp.**
MiCM	Chorismate mutase	Hormone and/or defense	–	Huang et al. (2005)
Mi8D05	Unknown	Transport	Tonoplast intrinsic protein AtTIP2	Xue et al. (2013)
MiCRT	Calreticulin	Defense	–	Jaouannet et al. (2013)
Mi16D10	CLE-like peptide	Transcription	Scarecrow-like transcription factor AtSCL6 and 11	Huang et al. (2006)
MiEFF1	Unknown	Unknown	–	Jaouannet et al. (2012)
MjNULG1	Unknown	Unknown	–	Lin et al. (2013)

## NEMATODE EFFECTORS HIJACK KEY CELLULAR FUNCTIONS

It remains unclear whether the nematode stylet perforates both the cell wall and the plasma membrane, to deliver effectors directly to the cytoplasm of the host cells. The apoplasm appears to be a major target of nematode effectors (Rosso et al., 2011; Vieira et al., 2011). However, nematode effectors may also be located within the host cells, where they may target different subcellular domains and assume highly diverse cellular functions (Haegeman et al., 2012; Hewezi and Baum, 2013). The first secreted proteins from PPNs to be characterized were cell wall-degrading and cell wall-modifying enzymes, such as β-1,4-endoglucanases, pectate lyases, polygalacturonases, and expansins, which are involved, in particular, in the invasion of root tissues by preparasitic juveniles and the migration of nematodes (Davis et al., 2011). These enzymes may also play an important role in FC formation, supporting the tremendous expansion of RKN-induced giant cells and facilitating syncytium formation. Effectors also target host enzymes to potentiate their function. Indeed, a *H. schachtii *(Hs) cellulose-binding protein, HsCBP, interacts with an *Arabidopsis *pectin methylesterase, potentially promoting the activity of this pectin-modifying enzyme or rendering cell wall polymers more accessible to other wall-degrading enzymes (Hewezi et al., 2008).

The *de novo* organogenesis underlying the construction of a nematode feeding site has a major impact on cell morphology and function as described above. Effectors mimicking plant compounds or binding to host proteins have been characterized. These molecules can affect plant signaling, hormone balance, and cell morphogenesis. The CNs secrete active CLAVATA3/ESR (CLE)-like proteins (Wang et al., 2005, 2011). In plants, CLE-like peptides play an essential role in meristem differentiation. These effectors seem to be secreted into the cytoplasm of host cells, from which they are transported to the plant apoplasm, where they mimic plant CLE signaling peptides and interact at the plasma membrane with leucine-rich repeat (LRR) receptor kinase family proteins, resulting in the formation and maintenance of syncytia (Guo et al., 2011). Another example of an effector having an impact on FC formation through the manipulation of host physiology is provided by Hs19C07, an effector that may modify hormone balance (Lee et al., 2011). Indeed, Hs19C07 interacts with the *Arabidopsis *plasma membrane auxin influx transporter LAX3, which modulates auxin influx in syncytia, thereby facilitating their development. Furthermore, both CNs and RKNs secrete proteins homologous to plant chorismate mutases (Bekal et al., 2003; Jones et al., 2003; Huang et al., 2005; Vanholme et al., 2009). The overexpression of nematode chorismate mutases *in planta* alters root growth (Doyle and Lambert, 2003), and it has been suggested that these effectors affect the auxin pool within the host cells. The recently characterized *M. incognita* effector Mi8D05 affects a different function of plant cells (Xue et al., 2013). Mi8D05 is essential for parasitism, as revealed by RNAi and overexpression approaches, and the overproduction of this effector strongly stimulates the growth of plant shoots. This effector has been shown to interact with a plant aquaporin tonoplast intrinsic protein (TIP2), suggesting a role in the regulation of solute and water transport within giant cells, promoting giant cell enlargement and nematode feeding.

Plants protect themselves against pathogen attacks through a combination of constitutive and induced strategies. The induction of plant defenses involves the recognition of compounds derived from the pathogen, called pathogen-associated molecular patterns (PAMPs). Pattern-triggered immunity (PTI) results from PAMP perception, leading to the activation of signaling pathways that restrict pathogen growth and promote host disease resistance (Jones and Dangl, 2006). No PAMPs have been described in nematodes, but secreted proteins and products of cell wall degradation may be recognized as such. Transcriptomic analysis has shown that a massive down-regulation of genes involved in plant defense is associated with the early stages of plant–nematode interaction (Jammes et al., 2005; Barcala et al., 2010; Damiani et al., 2012). This suggests that PPNs can suppress PTI. Various effectors that affect plant stress and defense responses have, indeed, been characterized (Smant and Jones, 2011). The nematode chorismate mutases mentioned above affect the plant shikimate pathway, thereby decreasing the synthesis of salicylic acid and phytoalexin through competition with chorismate, and preventing the triggering of host defense (Doyle and Lambert, 2003). Hs10A06 effector targets *Arabidopsis* spermidine synthase 2. Plants overproducing Hs10A06 are more susceptible to CNs and to bacterial and viral pathogens and produce smaller amounts of pathogenesis-related (PR) proteins. Hs10A06 acts on salicylic acid signaling and the antioxidant machinery, thereby protecting nematodes against plant defense responses (Hewezi et al., 2010). Similarly, the Hs4F01 annexin-like effector is secreted into the cytosol (Patel et al., 2010), where it interacts with an oxidoreductase of the 2OG-Fe(II) oxygenase family to prevent the triggering of host defense. Another CN effector, Hg30C02, interacts physically with a plant β-1,3-endoglucanase, a potential PR protein, and may thus be involved in defense suppression (Hamamouch et al., 2012). However, only *M. incognita* calreticulin (Mi-CRT), which suppresses defences induced by the PAMP elf18 when expressed *in planta,* has been shown to have a direct effect on PTI suppression (Jaouannet et al., 2013).

Plants have evolved resistance proteins that can recognize, either directly or indirectly, pathogen effectors, and induce effector-triggered immunity (ETI; Jones and Dangl, 2006). Several plant proteins conferring resistance to nematodes have been identified, mostly nucleotide-binding LRR (NB-LRR) proteins. However, very few nematode avirulence effectors have been identified (Smant and Jones, 2011). The CN effectors repertoire include a large family of secreted effectors containing a SPRY domain, named SPRYSECs (Jones et al., 2009; Rehman et al., 2009). One cytoplasmic *G. pallida* SPRYSEC (GpRBP-1) has been shown to be the avirulence target of the GPA-2 NB-LRR-resistant protein (Sacco et al., 2009). Remarkably, the GrSPRYSEC-19 protein has been shown to suppress the ETI mediated by several NB-LRR-resistant proteins, including GPA-2 (Postma et al., 2012). However, SPRYSEC-19 does not seem to mediate nematode resistance, despite interacting physically with SW5F, an SW5 NB-LRR-resistant protein from tomato (Rehman et al., 2009).

## NEMATODE EFFECTORS TARGET HOST CELL NUCLEI

The manipulation of host cell processes, such as the cell cycle, gene expression, and immunity, almost certainly involves the targeting of the host nucleus by secreted effectors. Bioinformatic analyses of predicted effectors have revealed the presence of nuclear localisation signals (NLSs) in several secreted proteins from both CNs (Gao et al., 2003; Elling et al., 2007) and RKN (Huang et al., 2003; Roze et al., 2008), potentially allowing nuclear import. Proteomic studies have identified 486 proteins secreted by *M. incognita*, 66 of which were found to have a putative NLS, or DNA-binding or chromatin-binding domains (Bellafiore et al., 2008).

The use of green fluorescent protein (GFP)-fusions in transient expression assays has confirmed the nuclear localization of some of these effectors within plant cells (Elling et al., 2007; Jones et al., 2009). For instance, MiEFF1 is a small secreted protein of 122 amino acids (aa), with no predicted homologs in databases and no known functional domain. It has a NLS and localizes to the nucleus when transiently expressed in tobacco cells (**Figure 1E**; Jaouannet et al., 2012). Interestingly, some effectors are found in the cytoplasm when their full-length forms are produced *in planta,* but their truncated forms have a nuclear or nucleolar distribution, suggesting that they may be relocalized after modification of the protein within the host cell (Tytgat et al., 2004; Elling et al., 2007). In this way, the putative ubiquitin extension protein HsUbiI is delivered to the host cell cytoplasm, and the cleavable C-terminal domain of the protein is directed to the nucleolus, where it may be involved in ribosome synthesis and parasitism (Tytgat et al., 2004).

Recently, immunolocalization approaches have shown two RKN effectors to be effectively delivered to giant cell nuclei (Jaouannet et al., 2012; Lin et al., 2013). Immunostainings demonstrate MiEFF1 is produced in the dorsal esophageal gland of the nematode and is secreted through the stylet into the giant cells, in which it is transported into the nuclei (**Figures 1F,G**; Jaouannet et al., 2012). Similarly, *M. javanica* MjNULG1a is a 274 aa protein of unknown function with two predicted NLS localizing in FC nuclei. Transgenic plants overproducing MjNULG1a are more sensitive to RKN, and RNAi studies *in planta* have provided evidence of a role for this protein in nematode parasitism (Lin et al., 2013). Both MiEFF1 and MjNULG1a seem to be specific to early steps in parasitism, but it remains unclear whether these effectors are involved in giant cell formation. Identification of the host cell targets of these proteins is underway and should shed light on their functions.

Very few host targets of nematode effectors that could form part of the host nuclear machinery corrupted to promote parasitism have been identified to date with yeast two-hybrid approaches. The *M. incognita* effector Mi16D10, which encodes a novel 13-amino acid secretory peptide, appears to be important for nematode development, as shown by RNAi approaches, and it favors root growth when produced *in planta*. Two plant SCARECROW-like transcription factors that interact with the Mi16D10 protein have been identified (Huang et al., 2003, 2006). In plants, these transcription factors play a key role in regulating root meristem identity and root development, and Mi16D10 may thus function in the extensive transcriptional reprogramming responsible for FC ontogenesis. The CN effector Hs10A07 contains a NLS, but is generally located in the cytoplasm when produced in plant cells (Elling et al., 2007). However, Hs10A07 is translocated from the cytoplasm to the nucleus following its interaction with a specific *Arabidopsis* protein kinase. Once inside the nucleus, this Hs10A07 effector interacts with transcriptional regulators and plays a role in parasitism (Hewezi and Baum, 2013). The Hs32E03 effector is located in the nucleus following the transient expression of its gene in plant cells (Elling et al., 2007). During parasitism, this effector interacts with nuclear proteins, leading to its localization in nuclear bodies (Hewezi and Baum, 2013). However, it remains unclear how this particular pattern of nuclear localization promotes parasitism. Finally, some SPRYSEC proteins localize to the nucleus of plant cells when transiently produced *in planta* (Jones et al., 2009), and bimolecular fluorescence complementation assay have confirmed that SPRYSEC-19 interacts strongly with the tomato SW5F-resistant proteins in infiltrated tobacco cell nucleoli (Postma et al., 2012). The putative function of this interaction remains unknown, but it does not appear to be involved in the ETI suppression mediated by SPRYSEC-19.

## CONCLUSION AND PERSPECTIVES

The identification of effectors is a major challenge in our understanding of the molecular aspects of plant–nematode interactions. Tremendous progress has been made toward the building of nematode effector repertoires since the completion of several genome sequencing. We still know little about the functions of these effectors and the host processes manipulated during the interaction to mediate the transformation of root cells into hypertrophied and multinucleate FCs. Functional characterization will be required to improve our understanding of the way in which these effectors promote host plant parasitism. Transformation procedures are currently lacking for PPNs, but such functional analysis should benefit from the recent development of RNAi approaches (Rosso et al., 2009), effector immunocytochemistry and the cellular imaging of feeding sites (Vieira et al., 2012a,b). A major breakthrough will result from identification of the plant targets of these effectors. Efforts to develop high-throughput approaches for such screening are already underway.

Host plant proteins targeted by effectors from many plant pathogenic microorganisms are being identified. It will be of particular interest to determine whether there are conserved parasitism strategies and whether nematodes and other plant pathogens target similar proteins. As obligate biotrophic parasites, nematodes must protect themselves against plant defenses and protect the host cells they need for feeding. PPNs may therefore target key components of the plant immune system corrupted during other plant–pathogen interactions. The GrVAP1 of CNs, Avr2 from the fungus *Cladosporium fulvum* and the EPIC1 and EPIC2B effectors from the oomycete *Phytophthora infestans* all target the same host papain-like cysteine protease, Rcr3^pim^ (Lozano-Torres et al., 2012), the tomato Cf-2 protein mediating resistance to *G. rostochiensis* and *C. fulvum* in a Rcr3^pim^-dependent manner.

Plant pathogens seem to target the plant nuclear machinery during infection (Bierne and Cossart, 2012; Caillaud et al., 2012; Deslandes and Rivas, 2012). Recent studies have shown that nematode effectors may indeed be localized to host nuclei or interact with host nuclear proteins. In addition, Hewezi and Baum (2013) have suggested that CN effectors recruit proteins involved in nucleocytoplasmic movement and nuclear dynamic during the parasitization of their hosts. These processes play an important role in several plant–pathogen interactions (Wiermer et al., 2007; Rivas, 2012). The identification of nematode effectors likely to bind DNA directly and affect host gene expression remains a major challenge. The molecular characterization of effectors and their plant targets is a key step toward understanding the factors determining nematode virulence, plant susceptibility or immunity and host range, and will open up new perspectives for controlling nematodes and other agronomically important pathogens.

## Conflict of Interest Statement

The authors declare that the research was conducted in the absence of any commercial or financial relationships that could be construed as a potential conflict of interest.
